# Asherman syndrome: Audit of a single‐operator cohort of 423 cases

**DOI:** 10.1111/ajo.13182

**Published:** 2020-05-26

**Authors:** Thierry Vancaillie, Karen Chan, Jinzhu Liu, Rebecca Deans, Elizabeth Howard

**Affiliations:** ^1^ Department of Women’s and Children University of New South Wales Sydney New South Wales Australia; ^2^ Women’s Health and Research Institute of Australia Sydney New South Wales Australia; ^3^ Department of Gynaecology Royal Hospital for Women Sydney New South Wales Australia; ^4^ Sydney Children’s Hospital Sydney New South Wales Australia; ^5^ Genea Sydney New South Wales Australia

**Keywords:** Asherman syndrome, infertility, intra‐uterine adhesion

## Abstract

**Background:**

The diagnosis of Asherman syndrome, or ‘intra‐uterine adhesions’ is often overlooked when the symptoms of amenorrhea and hematometra are missing.

**Aims:**

This audit reviews the clinical data of a large cohort of patients treated by a single operator.

**Materials and Methods:**

From July 1998 till the end of December 2017, 423 patients with intra‐uterine adhesions were treated by a single operator. Clinical information was obtained by review of the medical files and phone interviews.

**Results:**

Amenorrhea was recorded in 163/423 patients (38.5%), 225/423 (53.2%) patients did not have amenorrhea and for 35/423 (8.3%) patients the information was missing. A hematometra was documented in 19/423 (4.5%) patients. Pregnancy was achieved in 215/246 (87.4%). Patients with stage II disease did best with a pregnancy rate of 94.5% (*P* = 0.029).

**Conclusion:**

Asherman syndrome should be considered in any woman with a history of miscarriage or postpartum curettage who then fails to conceive again.

## Introduction

Asherman described a syndrome of traumatic amenorrhea. However, amenorrhea is not a necessary element. The term ‘intra‐uterine adhesions’ has been put forward as an alternative and is widely used in the fertility community.

The aim of this retrospective audit is to present the near‐20‐year experience of a single operator in treating Asherman syndrome and intra‐uterine scarring. Issues about obstetric outcome were addressed in collaboration with other colleagues from the department and are published elsewhere.^1^

## Materials and Methods

From July 1998 until the end of December 2017, 423 patients with intra‐uterine adhesions were treated by a single operator (TV). Ethics approval to contact the patients for completion of their records was obtained (SEHS LHD Ethics committee approval number 07/207 24). All patients presented with infertility, with the majority experiencing secondary infertility with at least one previous pregnancy. Twenty‐one out of 423 (5.0%) patients were nulliparous with surgical trauma or an infectious process as the presumed cause for the intra‐uterine scarring.

For longitudinal analysis of the data, patients were empirically grouped into five‐year blocks. The first block up to the end of 2004 also includes the few patients from before the turn of the century and the last block is only two years long (2016 + 2017). A minimum of one‐year follow‐up is available.

For staging of the condition, a system is used, adapted from the one originally described by Dr. Wamsteker.^2^, ^3^ His method of staging is based on evaluation of the proportion of the cavity affected by scarring using hysterosalpingography: stage I = <25% of the cervical canal and cavity affected; stage II = <50% of the cervical canal and cavity affected; stage III = <75% of the cervical canal and cavity affected; and stage IV = more than 75% of the cervical canal and cavity affected. One exception to this classification based on affected surface area is that when the internal cervical os is fully closed with or without extension into the cervical canal or the isthmus, the patient is classified as having at least stage II.

The surgical technique for treatment of the condition has been previously published.^4^, ^5^ Briefly, both X‐ray and trans‐abdominal sonography are used for guidance of the surgical procedure which consists of resection of scar tissue using hysteroscopic micro‐instruments (Karl Storz, Tuttlingen, Germany).

From July 2008 a hyaluronic acid emulsion was used to fill the cavity at the end of the procedure in an attempt to promote normal healing.^6^, ^7^ In October 2015, platelet‐rich plasma introduced into the uterus at the end of the procedure in patients 38 years or older with stage III or IV, was added to the treatment algorithm with the same intention.^8^

**Figure 1:**
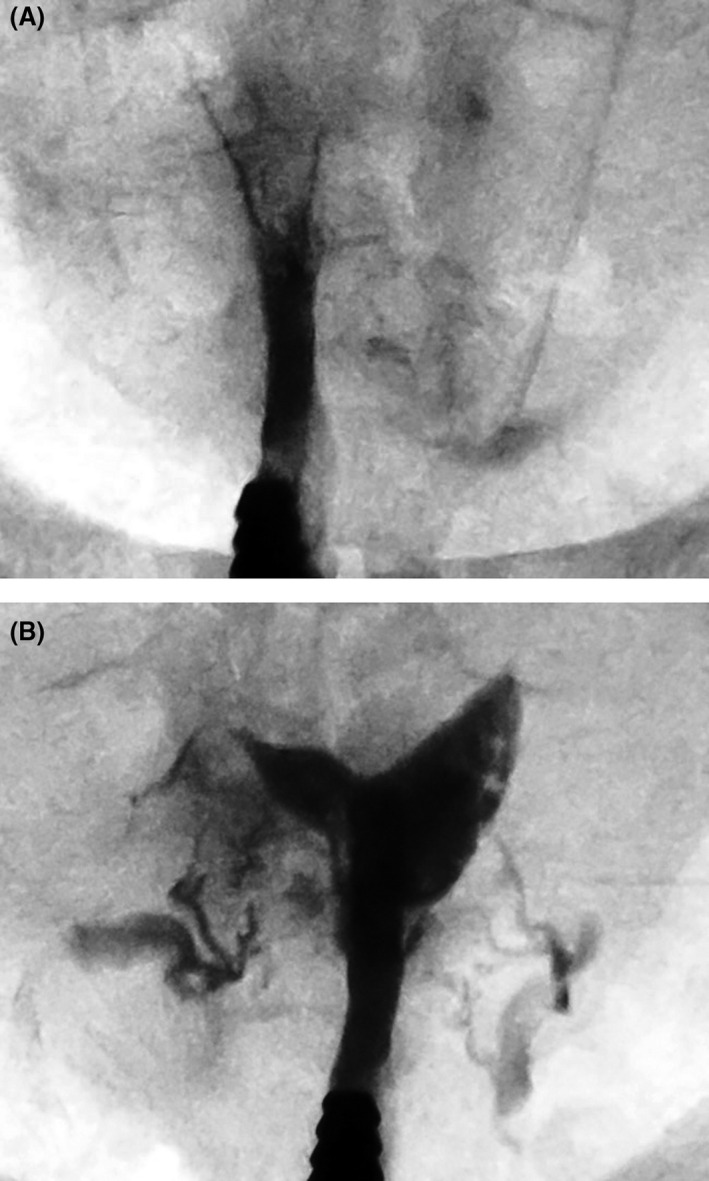
Xray images of a patient with stage III Asherman at the start (A) and the end (B) of the first of three procedures required to treat the condition.

Patients with stage I or II were allowed to attempt conception immediately after treatment. Those with stage III or IV were scheduled for a sonohysterogram if the procedure was deemed complete, or a repeat synechiolysis if the treatment was deemed incomplete. Some patients with stage III or IV underwent sequential procedures before being allowed to attempt conception.^9^ The number of procedures varied from two to five. Figure 1A shows the hysterosalpingogram of a patient with stage III disease during the early stages of the synechiolysis and Figure 1B shows the same patient at the end of the first procedure. A follow‐up procedure was scheduled in this particular patient.

Medical management consisted of doxycycline 100 mg daily for a total of seven days, starting two days prior to the planned surgery. In some cases, the patient’s menstrual cycle was manipulated to accommodate scheduling, using a standard combination oral contraceptive pill continuously until seven days prior to the scheduled procedure. All patients would take 2 mg of oestradiol orally starting on day two of menses and continue until ten days after intervention.

Pregnancy was defined as an ultrasound demonstrating a pregnancy regardless of location or viability.

Analyses were performed by descriptive statistics for clinical presentation and patient demographics and by analysis of variance (ANOVA), *t*‐test, χ^2^ tests and multivariate analysis for pregnancy outcome (SPSS, version 25, IBM, Armonk, NY, USA 2018, and R, version 3.6.2, r‐project.org, 2019).

## Results

### Age distribution

**Figure 2:**
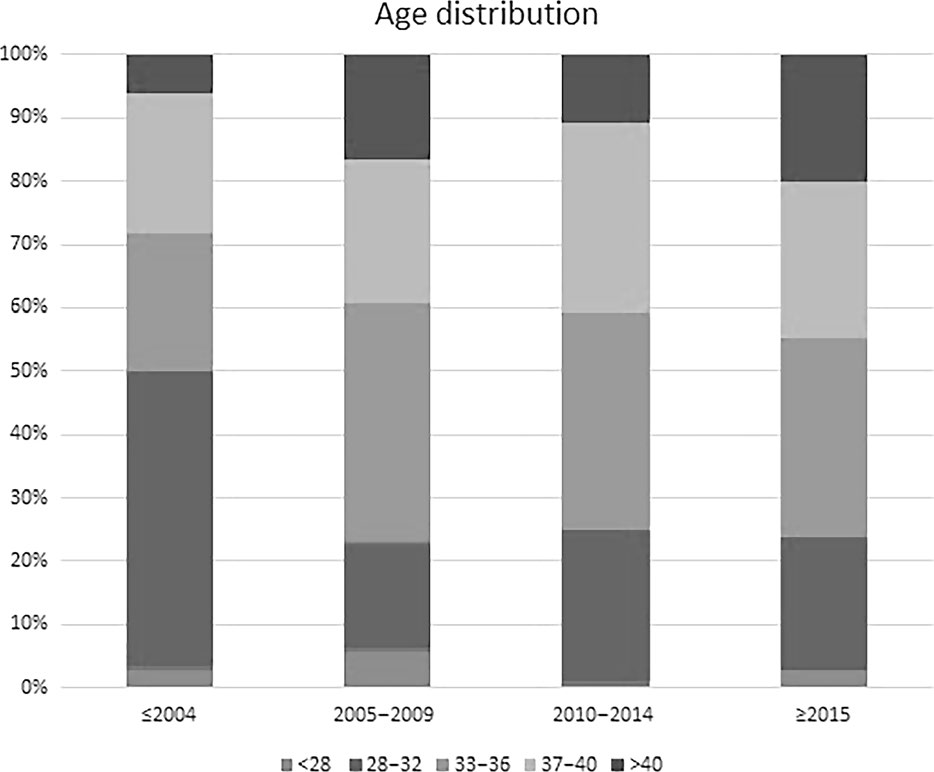
Age distribution of patients over time.

Figure 2 shows a bar graph of the age distribution of patients within each time period.

The average age of the first block of patients (*n* = 38) was 33.9, the average age of the last block (*n* = 133) was 36.7 (*P* = 0.008; *t*‐test). Correlation between age and staging of the condition did not show any statistically significant trend (*P* = 0.238; one‐way ANOVA). There were no patients under the age of 28 in the stage IV group.

### Clinical presentation

The amenorrhea, dysmenorrhoea and hematometra rates are listed in Table 1

**Table 1:** Clinical presentation

	Amenorrhea	%	Dysmenorrhoea	%	Hematometra	%
Present	163	38.5	101	23.8	19	4.5
Absent	225	53.2	261	61.8	273	64.5
Missing data	35	8.3	61	14.4	131	31.0
Total	423	100	423	100	423	100

The incidence of amenorrhea, dysmenorrhoea (increased or de novo) and hematometra within the entire cohort. The relatively high number of missing data for the presence of hematometra is due to the fact that sonography is not routinely performed prior to treatment.

### Initiating event

Information on a potential trigger for adhesion formation is available in 386 of the 423 (91.3%) patients (Table 2).

**Table 2:** Trigger events

	*n*	%
Miscarriage	210	49.6
Postpartum intervention	102	24.1
Hysteroscopic surgery	27	6.4
Laparotomy + myomectomy	5	1.2
B Lynch suture (or similar)	4	0.9
Other (eg uterine embolisation)	7	1.7
Multiple triggers	31	7.3
Missing data	37	8.8
Total	423	100

Prevalence of events thought to be responsible for scar formation. In some cases, the trigger event was not clear or there were several potential candidate incidents. These cases were grouped in the category of multiple triggers.

The average delay between the initiating event and the presentation for management of 226/423 cases with available information, was 16.1 months. Patients with stage IV had a longer delay of 31.2 months (*P* < 0.01; *t*‐test). Patients who had been surgically treated elsewhere, unsuccessfully, prior to referral, had an average of ten months longer delay prior to treatment (23.0 months vs 13.3 months; *P* < 0.001; *t*‐test).

### Pregnancy rate

Conception outcome is available for 246 of the 424 (58.0%) cases. Pregnancy was achieved in 215 of these 246 women, representing an overall success rate of 87.4%. If all missing data are assumed to represent failures, the success rate was 50.8%. The pregnancy rate over time (Table 3) increased from 81.5% in 2004 and 80.0% in 2009, to 95.2% in 2014 (*P* = 0.037; χ^2^ test). The pregnancy rate for the last cohort is 84.8%, but there is a shorter observation time for them.

**Table 3:** Pregnancy† rate per block of time

	*n*	%
2004	22/27	81.5
2005–2009	44/55	80.0
2010–2014	80/84	95.2**
2015–2017	56/66	84.8

There are 14 files with inadequate information on the date of conception post‐treatment. The total number of patients available for analysis is therefore 232.

† Pregnancy is defined as an ultrasound demonstrating a pregnancy regardless of location or viability.

** *P* = 0.037 (χ^2^ test).

Those successful in achieving conception presented with an average age of 34.73 years old vs 36.99 for those who were unsuccessful (*P* = 0.011; *t*‐test). Patients with stage II Asherman experienced the best outcome with a conception rate of 94.5% (*P* = 0.039; χ^2^ test) (Table 4).

**Table 4:** Pregnancy† rate per stage of disease

Stage	*n*	%
1	46/54	85.2
2	86/91	94.5**
3	61/71	85.9
4	20/27	74.1
Total	213/243	87.7

The staging of the condition was unclear from the notes in three cases. The total number of patients with available staging of the condition therefore is 243.

† Pregnancy is defined as an ultrasound demonstrating a pregnancy regardless of locations or viability.

** *P* = 0.039 (Pearson’s χ^2^ test comparing stage II to the three other stages).

A generalised mixed effects regression model was used to determine the odds ratio of pregnancy for each of the Asherman syndrome stages after adjusting for maternal age and the number of corrective interventions, keeping participant ID as a random effect.

The estimates of odds ratios are relative to women with stage I Asherman syndrome. Women with stage II Asherman syndrome had an increased odds of becoming pregnant of 2.07 (95% CI: 1.11–3.84) times that of women with stage I; stage III patients had an increased odds of becoming pregnant of 1.79 times that of stage I; however, this was not statistically significant (95% CI: 0.93–3.46). There was a statistically significant association between maternal age and the odds of becoming pregnant, which decreased by a multiple of 0.92 (95% CI: 0.87–0.98) per year of age. There was no clinical or significant association between the number of interventions and the odds of a woman with Asherman syndrome becoming pregnant (1.01; 95% CI: 0.87–1.18).

The conception rate for those patients who developed intra‐uterine adhesions secondary to triggers, unrelated to pregnancy (32/386 patients with documented trigger event), is only 43.8%.

## Discussion

This retrospective audit has significant limitations due to the difficulty in collecting follow‐up data. However, the focus of this audit has been the clinical presentation and its evolution over time.

The data from this cohort show there is no typical presentation of Asherman syndrome. Amenorrhea despite ovulation, was present in less than half the patients. Too often this leads to a delay in diagnosis. The average delay in this cohort is 16 months, part of which can be explained by breastfeeding‐induced physiologic amenorrhea.

The increasing age of the Asherman patients reflects the general trend of delaying pregnancy in Australia. However, the ageing Asherman population does not present with a more advanced stage of the condition. On the other hand, younger patients are more likely to be successful in achieving pregnancy after treatment. Therefore, age is an important factor in attaining success.

Patients developing intra‐uterine scarring after a miscarriage (first and second trimester) vs within the postpartum, have the same chance of achieving success after treatment. However, it is our impression that scarring more likely will involve the corpus after a postpartum trigger, whereas the isthmus is the more likely location after a miscarriage. Dealing with isthmic synechiae is technically less challenging.

The surgical technique with hysteroscopic micro‐instruments, the use of X‐ray, the addition of hyaluronic acid or platelet‐rich plasma, have not been tested via prospective randomised controlled trials. Results and discussions should be accordingly interpreted.

Patients with stage II Asherman achieve the best result (94.5%), surpassing the outcome of treatment of stage I (85.2%). The impact of intra‐uterine scarring on fertility is more complex than our current understanding. Stage II Asherman cases represent those patients with 50% of surface affected or less, but also those patients with occluded internal os, yet when the total affected area is <25%. The presence of scar tissue within the isthmus, even if the internal os is not completely closed, will induce amenorrhea in most cases without the development of a hematometra. The presence of a hematometra is uncommon (4.5%). It is therefore hypothesised that the presence of intra‐uterine scar tissue impedes normal endometrial function.

It is likely that women with intra‐uterine scarring, but with preserved endometrial function are fertile and may achieve conception. However, the question remains whether a pregnancy conceived under those circumstances is at a higher risk for miscarriage.^10^ The stage I group likely includes patients in whom the presence of minimal scar tissue is mostly an incidental finding and infertility is due to other factors.

The low conception rate (43.8%) in patients who developed intra‐uterine scarring secondary to a trigger unrelated to pregnancy is in line with other publications.^11^

**Figure 3:**
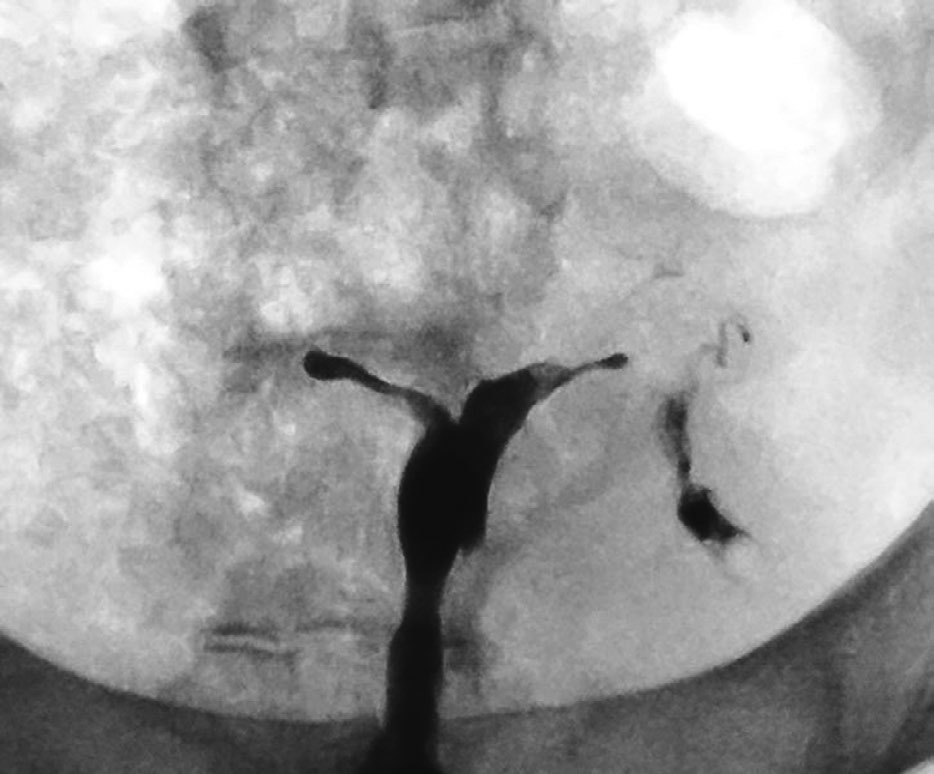
Xray image showing the imprint of a levonorgestrel intra‐uterine device, which had been placed after synecholysis at a different institution, several weeks after removal of the device.

Surgical management of intra‐uterine scarring requires expertise. In the interest of improved patient outcomes, an argument should be made for pooling of cases into dedicated treatment centres. An intra‐uterine contraceptive device or a custom‐made balloon are traditionally inserted^12^, ^13^, ^14^ as a mechanical barrier at completion of a synechiolysis.^15^ We choose not to use a true physical barrier. Our treatment philosophy is simple: (i) identify the scar tissue; (ii) remove the scar tissue; and (iii) promote normal healing. Figure 3 shows the hysterosalpingography of a patient treated elsewhere for removal of intra‐uterine adhesions in whom a levonorgestrel containing device was inserted. The X‐ray was taken several weeks after the device was removed, at the start of the first of three procedures this patient required to restore the uterine cavity. The phantom outline of the device is clearly identifiable with near complete obliteration of the surrounding uterine cavity. It is clear that insertion of the device did not achieve its intended goal.

The addition of hyaluronic acid emulsion was intended to improve the outcome of patients, especially those with stages III and IV. However, a significant improvement was not achieved.

In conclusion, intra‐uterine adhesions are not always associated with clinical symptoms. However, their presence should be considered in any woman presenting with infertility after a miscarriage or postpartum curettage. Treatment should be performed by experienced operators in dedicated centres. The surgical method used, namely endoscopic mechanical micro‐instruments under direct vision as well as sonographic and image intensifier control, yields good results.
